# Supplementary material for: Reprogramming of pancreatic adenocarcinoma immunosurveillance by a microbial probiotic siderophore
Source: Commun Biol. 2022 Nov 4;5:1181. doi: 10.1038/s42003-022-04102-4 (PMC9636404; doi:10.1038/s42003-022-04102-4)
Supplement: Supplementary file 2 — Supplementary Information [file 42003_2022_4102_MOESM2_ESM.pdf]

Supplementary Information

Reprogramming of Pancreatic Adenocarcinoma Immunosurveillance by a Microbial Probiotic Siderophore

Chaib et el.

A. List of Content:

B. Supplementary Figures 1-12

C. Supplementary Tables

D. Supplementary full scans of gels

Supplementary Figure 1

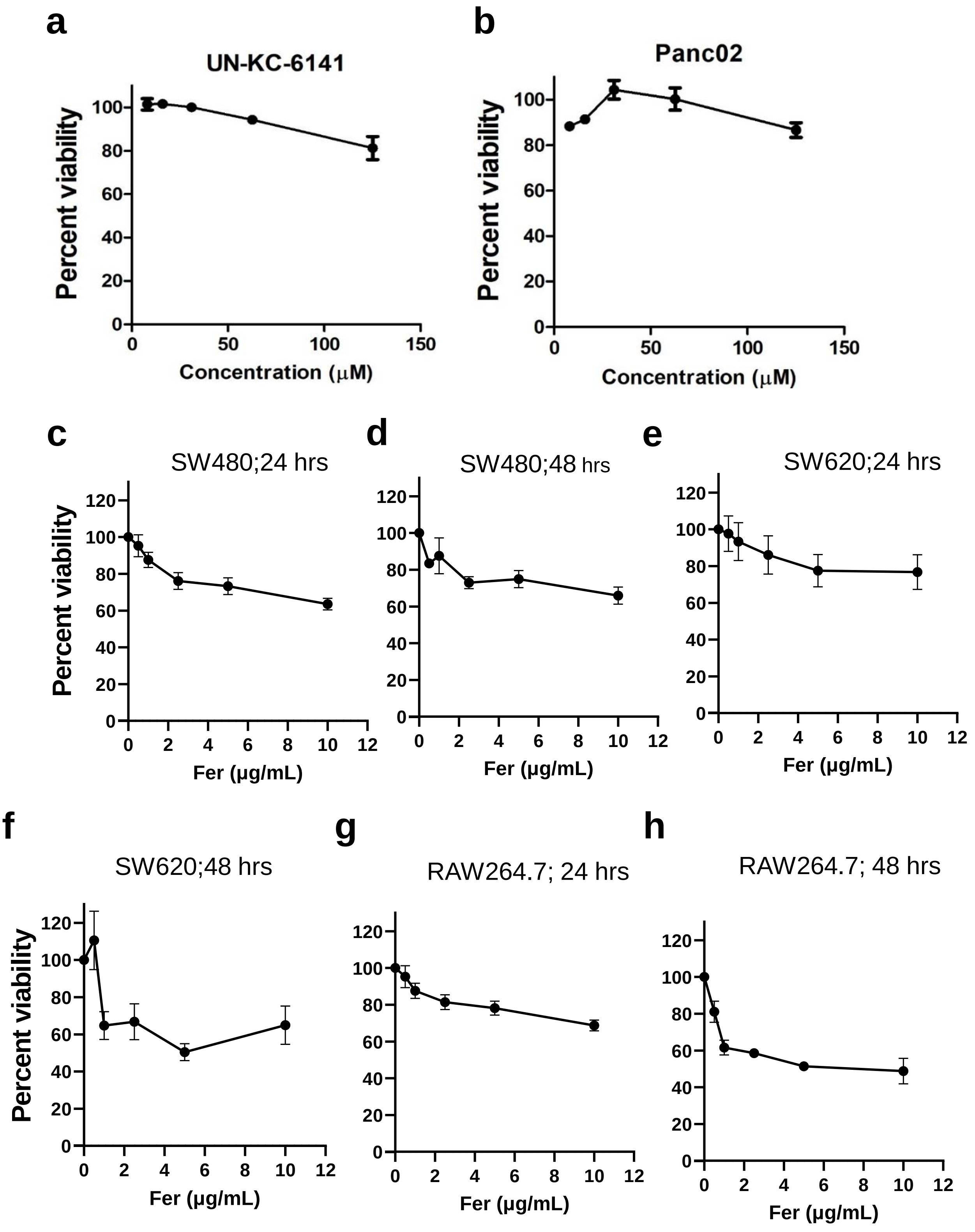

Supplementary Figure 1: Ferrichrome does not show direct cytotoxic effect on pancreatic cancer cells and murine macrophages. (a) and **(b)** Effect of ferrichrome of cell viability of two murine pancreatic cancer cell lines (UN-KC-6141 and Panc02) as determined by MTT assay for 48 hours. (**c-f**) Proliferation of colorectal cells SW480 (c-d) and SW620 (e-f) were examined by MTT at 24 and 48 hour as indicated. (**g-h**) RAW264.7 macrophage cell line proliferation was examined by MTT and 24 and 48 hours. Values shown as Mean ± SEM.

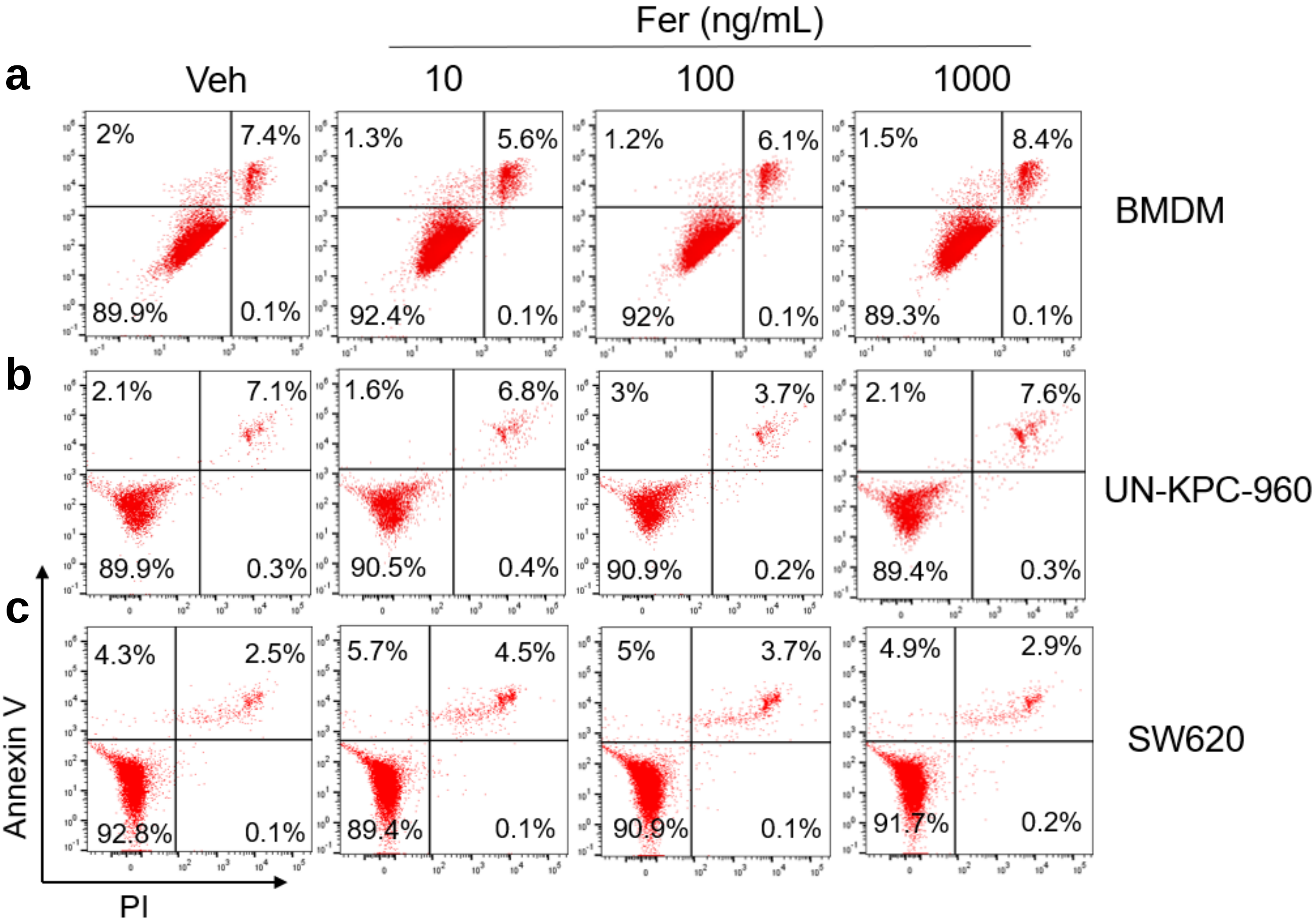

Supplementary Figure 2: Ferrichrome does not induce apoptosis in cancer cells or murine macrophages. Apoptosis of **(a)** murine bone marrow-derived macrophages (BMDM), **(b)** murine pancreatic cancer cell line (UN-KPC-960), and **(c)** human colon cancer cell line (SW620), and was determined by AnnexinV and propidium iodide (PI) staining after Ferrichrome (Fer) treatment for 24 hours at indicated doses compared to vehicle control (Veh). Values shown as Mean of N=3 biological replicates for BMDMs or technical replicates for cancer cell lines.

A.

Tube No.	Sample	OD 1	OD 2	OD 3	Average OD	Change in Absorbance	Endotoxin Concentration (EU/ml)
**1.**	LAL reagent water (Blank)	0.051	0.05	0.052	0.051	0	-
**2.**	0.1 EU/ml Standard	0.586	0.584	0.591	0.587	0.536	0.1
**3.**	0.05 EU/ml Standard	0.369	0.369	0.369	0.369	0.318	0.05
**4.**	0.025 EU/ml Standard	0.198	0.167	0.172	0.179	0.128	0.025
**5.**	0.01 EU/ml Standard	0.1	0.101	0.1	0.100333	0.049333	0.01
**6.**	Ferrichrome	0.095	0.093	0.093	0.093667	0.042667	**0.0074**

B.

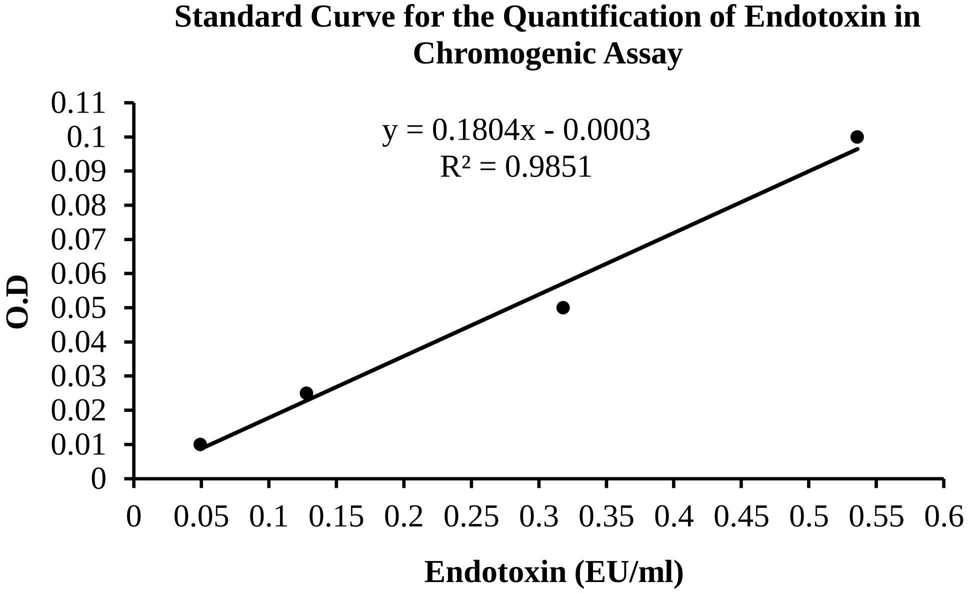

<u>**Supplementary Figure 3:**</u> **Ferrichrome stock is not contaminated by endotoxin.** LPS detection kit (GeneScript L00350C) was used to test the Ferrochrome batch lot used in this manuscript. N=3 technical replicates per dose. Endotoxin concertation in Ferrochrome was recorded **0.0074 EU/ml** which was below the lowest standard dose of endotoxin **(a-b)**.

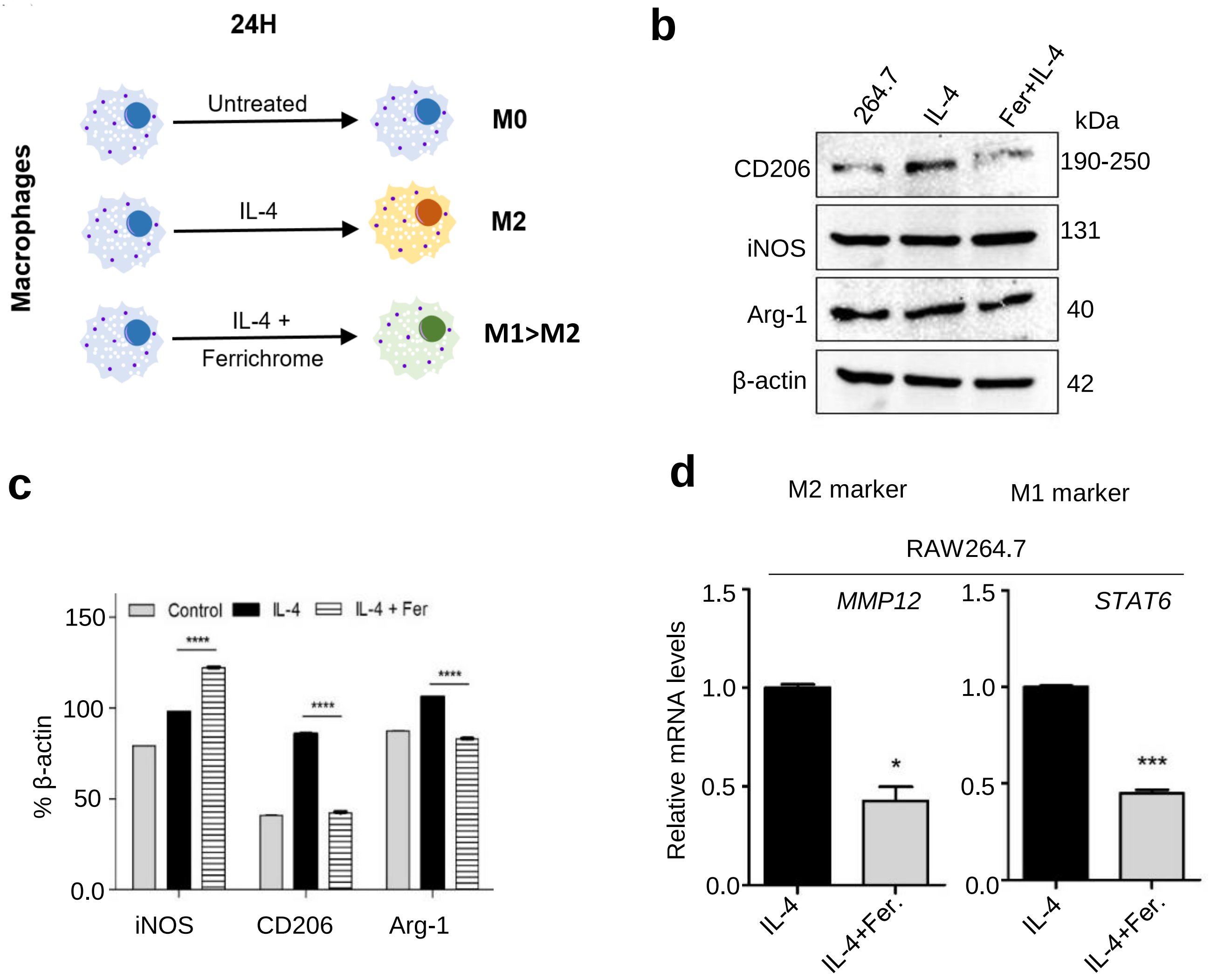

Supplementary Figure 4: Ferrichrome skews macrophage polarization toward M1-like phenotype, decreases expression of *Mmp12* and *Stat6* genes in murine macrophages. **(a)** Schematic representation of *in vitro* experimental design as in Figure 2. **(b)** Western immunoblot analysis of protein levels of M1 (iNOS) and M2 (CD206 and Arg1) expression in RAW 264.7 macrophages treated as explained in (A). **(c)** Quantification of Western immunoblot analysis of indicated proteins. **(d)** qPCR analysis of *Mmp12* and *Stat6* gene expression in RAW 264.7 cells treated with ferrichrome or vehicle in the presence of IL-4 for 24 hours. Mean ± SEM shown. $^*p < 0.05$ indicates statistical significance, as determined by Student’s t-test.

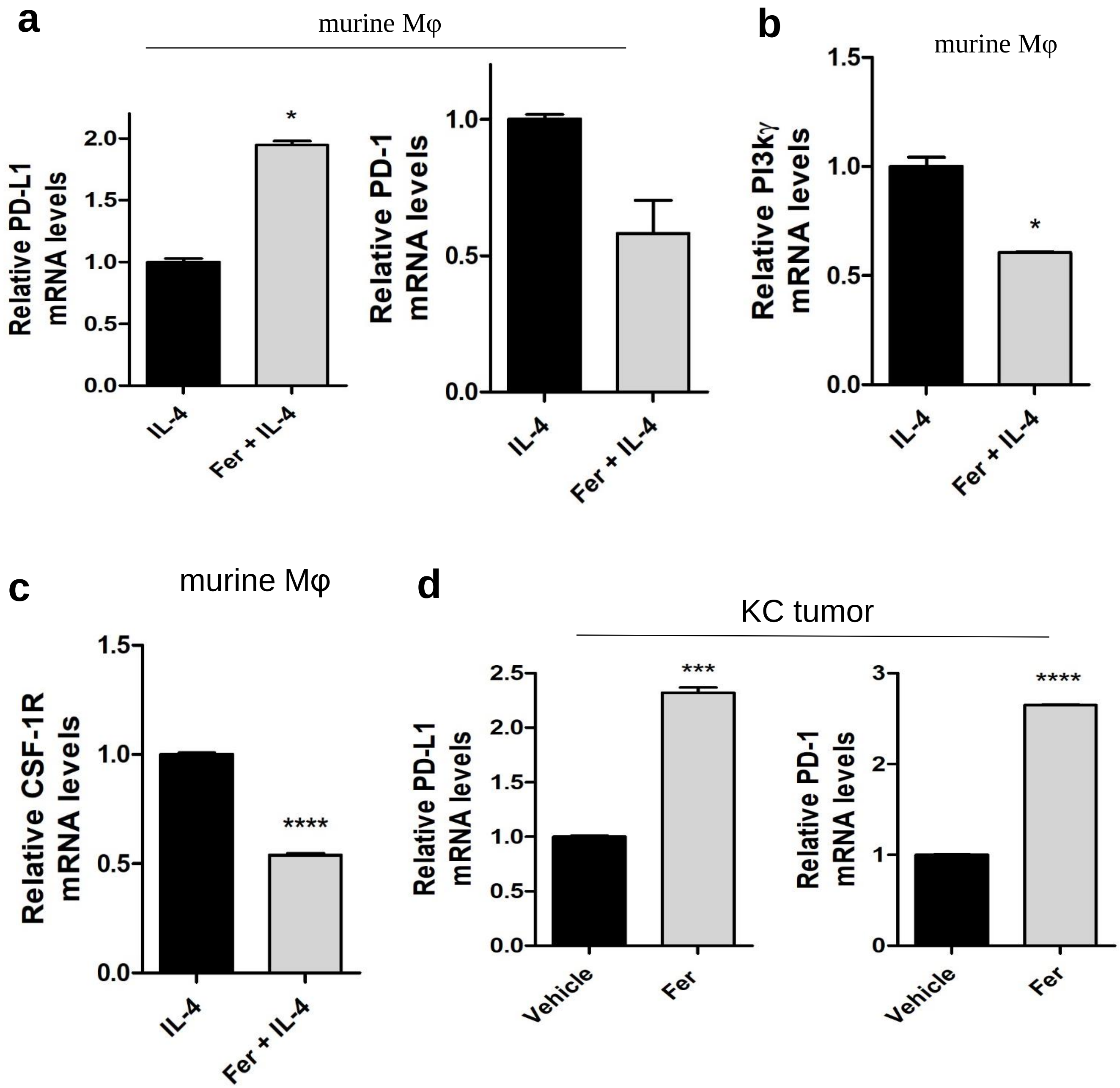

Supplementary Figure 5: Effect of ferrichrome on PD-1, PD-L1, CSF-1R, and PI3Kγ gene expression on macrophages in *in vitro* and in KC tumors *in vivo*. qPCR analysis of **(a)** PD-L1 and PD-1, **(b)** PI3Kγ gene expression in peritoneal macrophages treated with ferrichrome or vehicle in the presence of IL-4 for 24h (n=2). **(c)** CSF-1R and **(d)** qPCR analysis of PD-1 and PD-L1 gene expression in tumors treated with ferrichrome or vehicle (n=3 mice). Mean ± SEM shown. $*p < 0.05$ indicates statistical significance, as determined by Student's t-test.

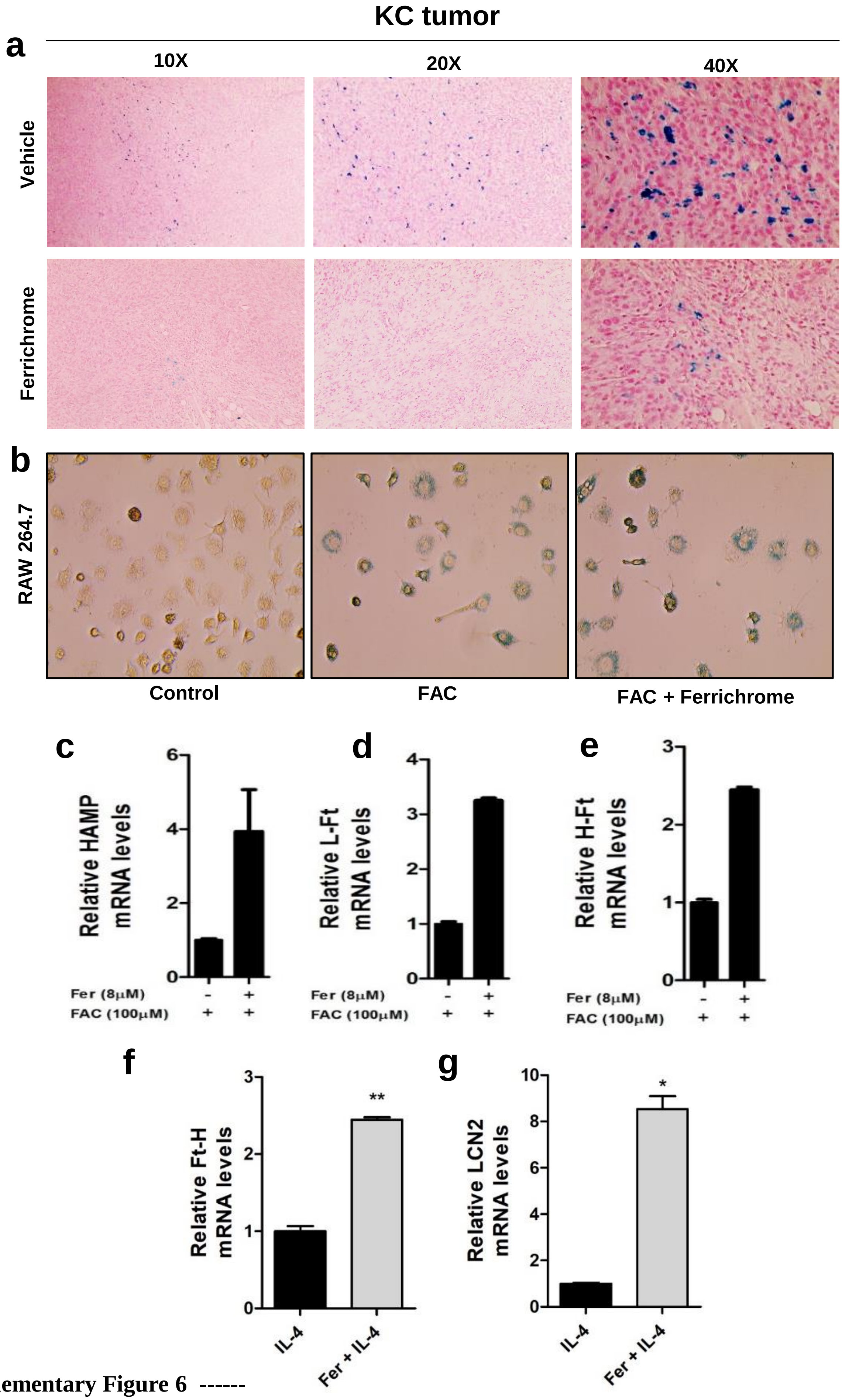

Supplementary Figure 6 ------

Supplementary Figure 6:

Ferrichrome modulates iron metabolism in macrophages by downregulating Fpn expression in a TLR4-dependent manner. **(a)** Iron staining of tumor sections treated with ferrichrome or vehicle (n=4 mice). **(b)** Iron staining of RAW 264.7 pre-treated with FAC (100 µM) in the presence or absence of ferrichrome for 8 hours. qPCR analysis of iron regulation genes **(c)** HAMP, **(d)** L-Ft and **I** H-Ft in RAW 264.7 cells pre-treated with FAC and treated with ferrichrome or vehicle for 24H. qPCR analysis of **(f)** Ft-H, **(g)** LCN2 and **(h)** Fpn gene expression in Raw 264.7 cells treated with ferrichrome or vehicle in the presence of IL-4 for 24H. **(i)** qPCR analysis of Fpn gene expression in BMDM isolated from WT or TLR4 KO mice and treated with vehicle or ferrichrome for 24H (n=2 mice). qPCR analysis of F gene expression in RAW 264.7 treated with different combinations of **(j)** FeSO4, LPS, ferrichrome and CLI-095; and **(k)** Fac and ferrichrome for 24 hours. **(l-o)** qPCR analysis of M1 genes in RAW 264.7 cells treated with LPS and pre-treated with CLI-095 for 24 hours. All these experiments have been repeated at least twice with similar results. Mean ± SEM shown. $^*p < 0.05$ indicates statistical significance, as determined by Student's t-test.

Supplementary Fig. 7

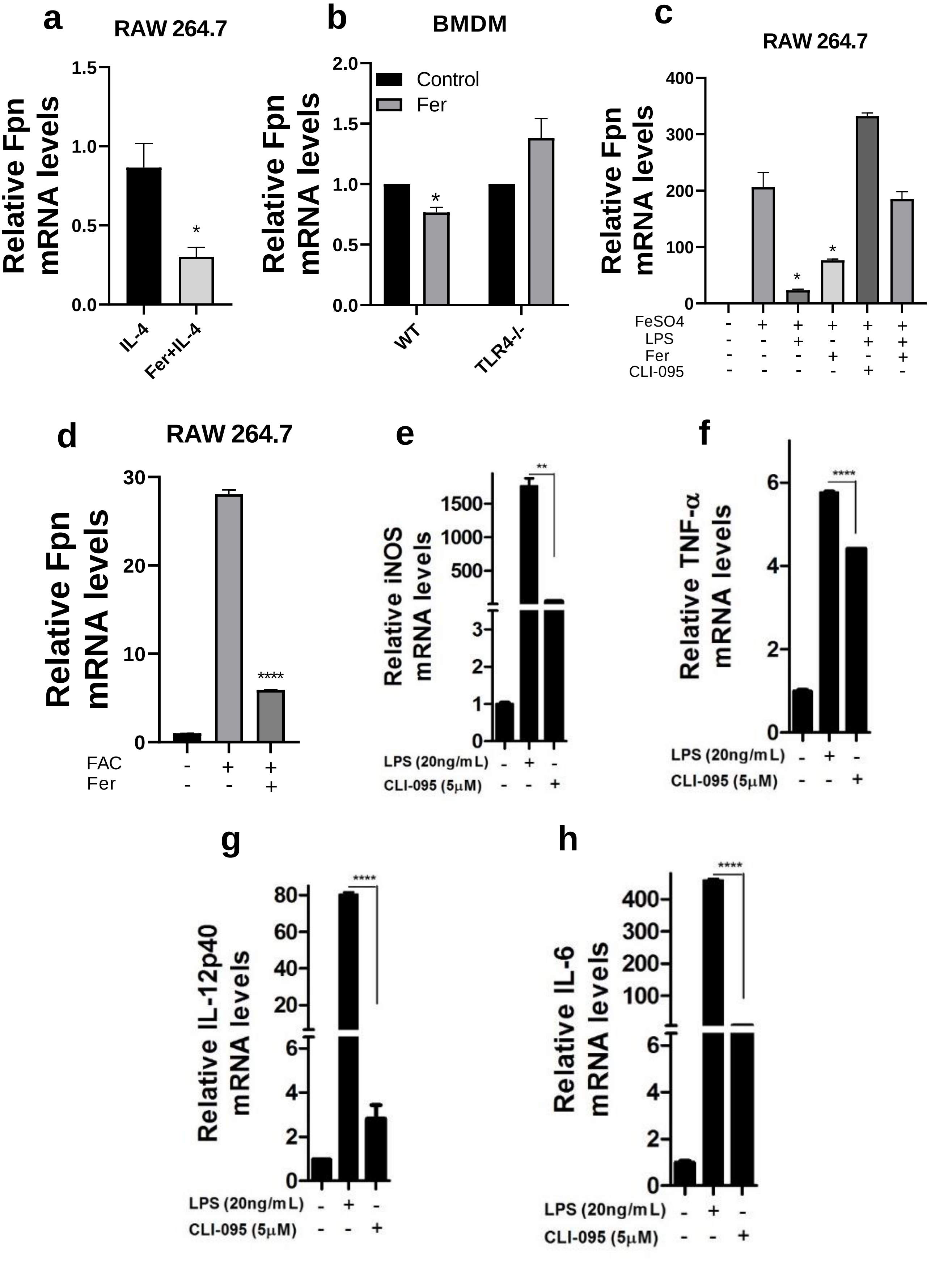

Supplementary Figure 7: Effect of ferrichrome on the expression of fpn and M1 and M2 markers in wild type and TLR4-/- mice macrophages. (a) Fpn gene expression in Raw 264.7 cells treated with ferrichrome or vehicle in the presence of IL-4 for 24h. **(b)** qPCR analysis of Fpn gene expression in BMDM isolated from WT or TLR4 KO mice and treated with vehicle or ferrichrome for 24H (n=2 mice). qPCR analysis of F gene expression in RAW 264.7 treated with different combinations of **(c)** FeSO4, LPS, ferrichrome and CLI-095; and **(d)** Fac and ferrichrome for 24 hours. **(e-h)** qPCR analysis of M1 genes in RAW 264.7 cells treated with LPS and pre-treated with CLI-095 for 24 hours. All these experiments have been repeated at least twice with similar results. Mean ± SEM shown. *$p < 0.05$ indicates statistical significance, as determined by Student's t-test.

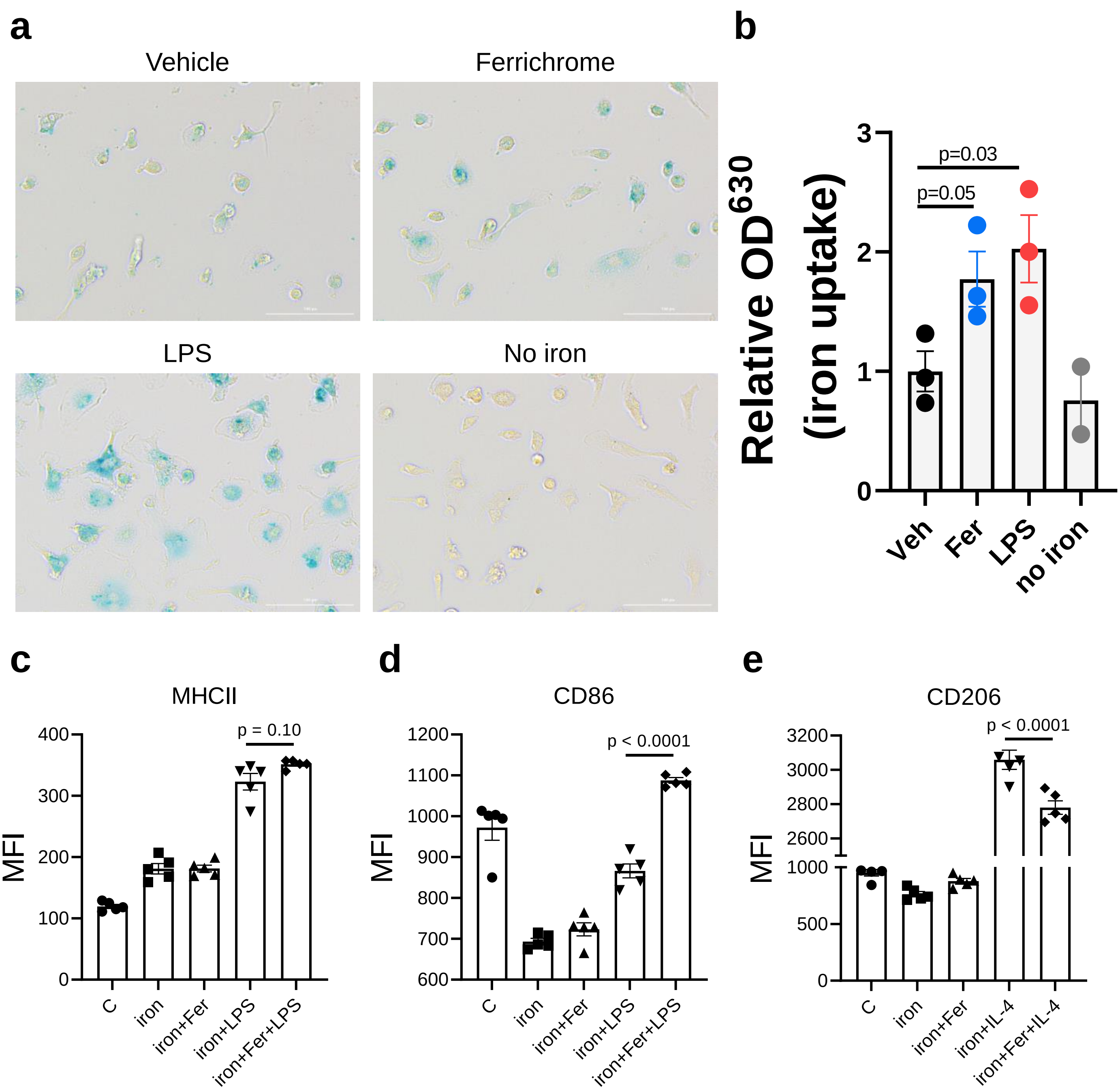

Supplementary Figure 8: Effect of ferrichrome on iron content and phenotype in BMDMs. BMDMs were treated with vehicle, ferrichrome or LPS (positive control) for 48h, then cells were washed with cold PBS twice before FeSO4 (500µM) supplemented media was added for 16h. **(a)** representative images of iron stain (blue) using ion stain kit (Prussian blue) as taken by bright field microscope. **(b)** Intracellular iron quantification as determined by optical density (OD) at 630nm relative to vehicle-treated cells. Negative control consisted of BMDMs supplemented with FeSO4-free media (no iron). C-E BMDMs were first loaded with iron as above for 16h, then cells were washed twice with cold PBS and ferrichrome, LPS or IL-4 were added for an additional 24h. Flow cytometry analysis was used to quantify the Mean Fluorescence Intensity of **(c)** MHCII, **(d)** CD86 and **(e)** CD206. Mean ± SEM shown for N=3 biological replicates for **(a, b)** and N=5 biological replicates for **(c-e)**. *$p < 0.05$ indicates statistical significance, as determined by Student's t-test.

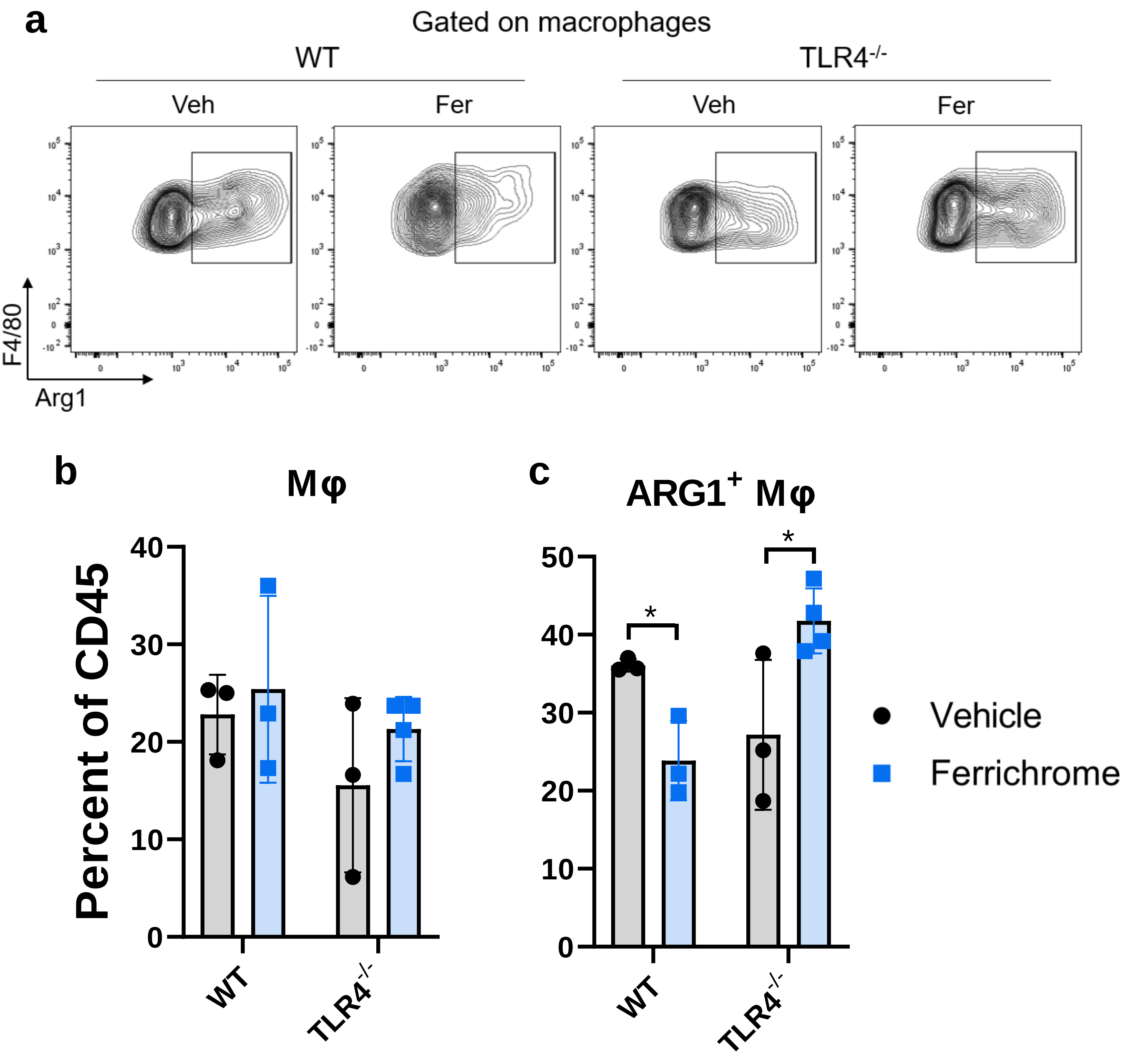

Supplementary Figure 9: Ferrichrome decreases M2-like macrophages *in vivo* in a TLR4-dependent manner. WT and TLR4$^{-/-}$ mice were injected with 10^6 KPC cells subcutaneously as previously described. Ferrichrome was administered IT on days 10, 11 and 12 before tumors were analyzed for macrophage content via flow cytometry analysis. **(a)** Representative flow cytometric plot of Arg-1+ macrophages in KPC tumors. **(b)** Total macrophage content and Arg1+ macrophage content in KPC tumors of WT and TLR4-/- mice treated with ferrichrome or vehicle. Mean ± SEM shown. *$p < 0.05$ indicates statistical significance, as determined by One-way anova. N=3-4 biological replicates.

Supplementary Fig. 11

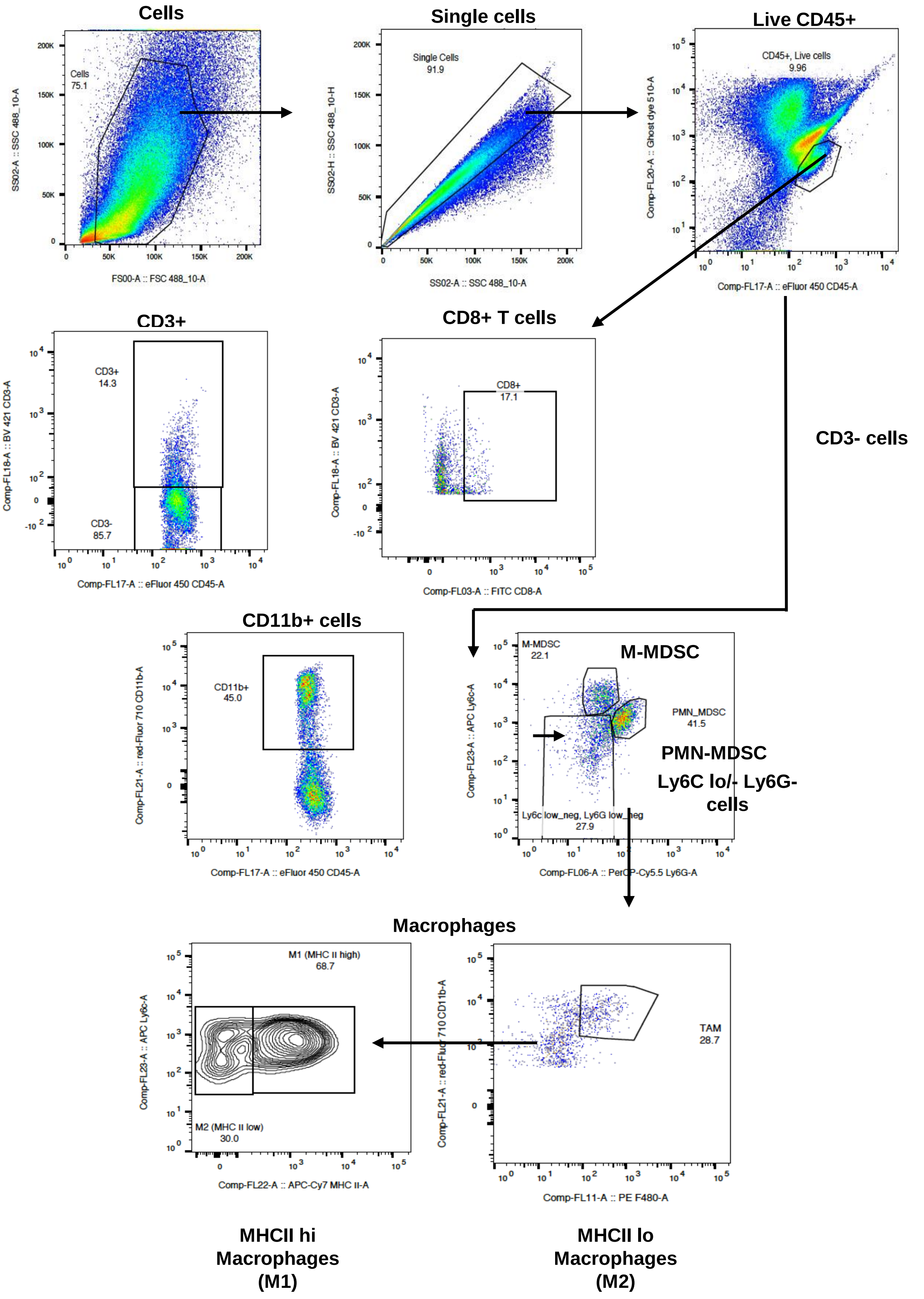

Supplementary Figure 10

Gating strategy for flow cytometry analysis of tumor infiltrating immune cells in UN-KC-6141 tumors. After gating total cells by plotting forward scatter versus side scatter areas, single cells by plotting side scatter height versus side scatter area and live CD45+ cells by plotting CD45 versus Ghost viability dye, immune cells were gated as follows:

Total T cells: (CD45+ CD3+); CD8+ T cells: (CD45+ CD3+ CD8+); myeloid cells: (CD45+ CD3- CD11b+); M-MDSC (CD45+ CD3- CD11b+ Ly6C-high Ly6G-); PMN-MDSC (CD45+ CD3- CD11b+ Ly6C-low Ly6G+); macrophages: (CD45+ CD3- CD11b+ Ly6C- Ly6G- F4/80+); M1-like TAM (CD45+ CD3- CD11b+ Ly6C- Ly6G- F4/80+ MHCII-high); M2-like TAM (CD45+ CD3- CD11b+ Ly6C- Ly6G- F4/80+ MHCII-low/-).

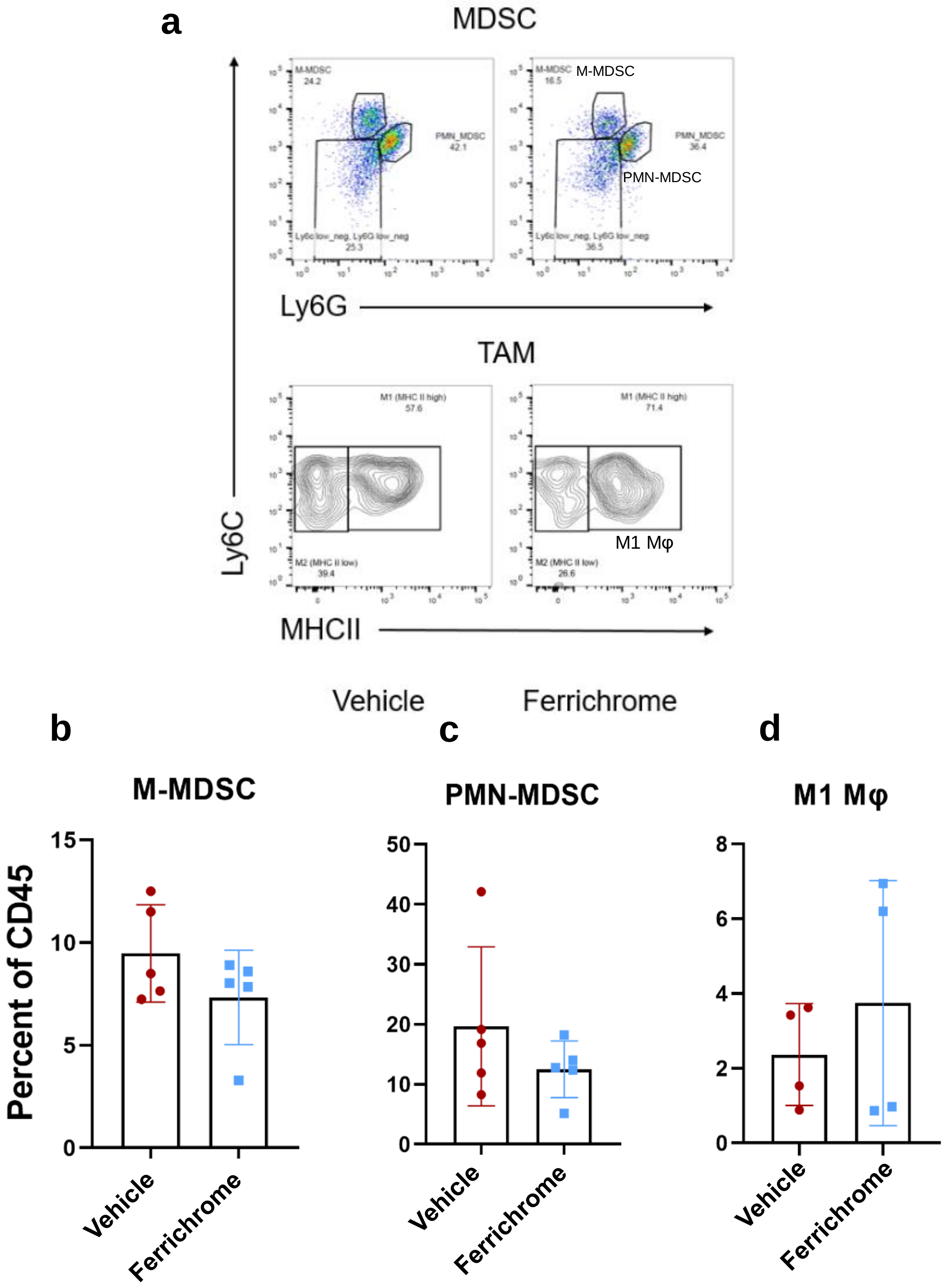

Supplementary Figure 11: Ferrichrome decreases MDSCs and increases M1 macrophage frequencies in pancreatic tumors. (a) Representative flow cytometric plot of PMN-MDSC, M-MDSC M1 and M2 TAM in ferrichrome or vehicle-treated tumors (n=4-5 mice). Frequency of **(b)** M-MDSC, **(c)** PMN-MDSC, **(d)** M1-like macrophages in UN-KC-6141 tumors treated with ferrichrome or vehicle.

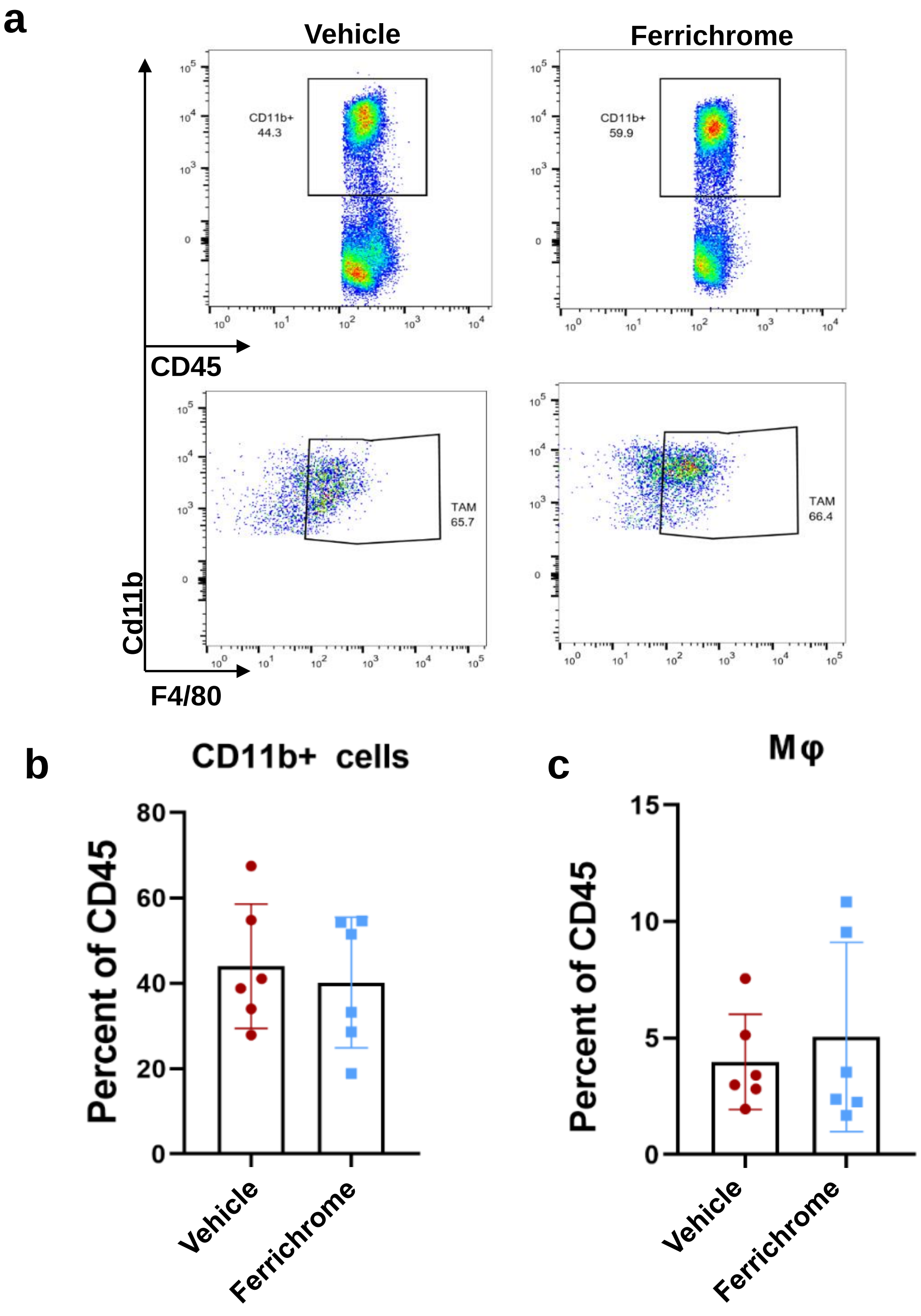

Supplementary Figure 12: Flow cytometry analysis of tumor infiltrating myeloid cells. **(a)** Representative flow cytometric plot of total myeloid cells and total macrophages (TAM) in ferrichrome or vehicle-treated tumors (n=6 mice). **(b)** Frequency of total CD11b+ cells and **(c)** total macrophages in tumors treated with ferrichrome or vehicle. Mean ± SEM shown. $*p < 0.05$ indicates statistical significance, as determined by Student's t-test.

D. Supplementary full scans of gels

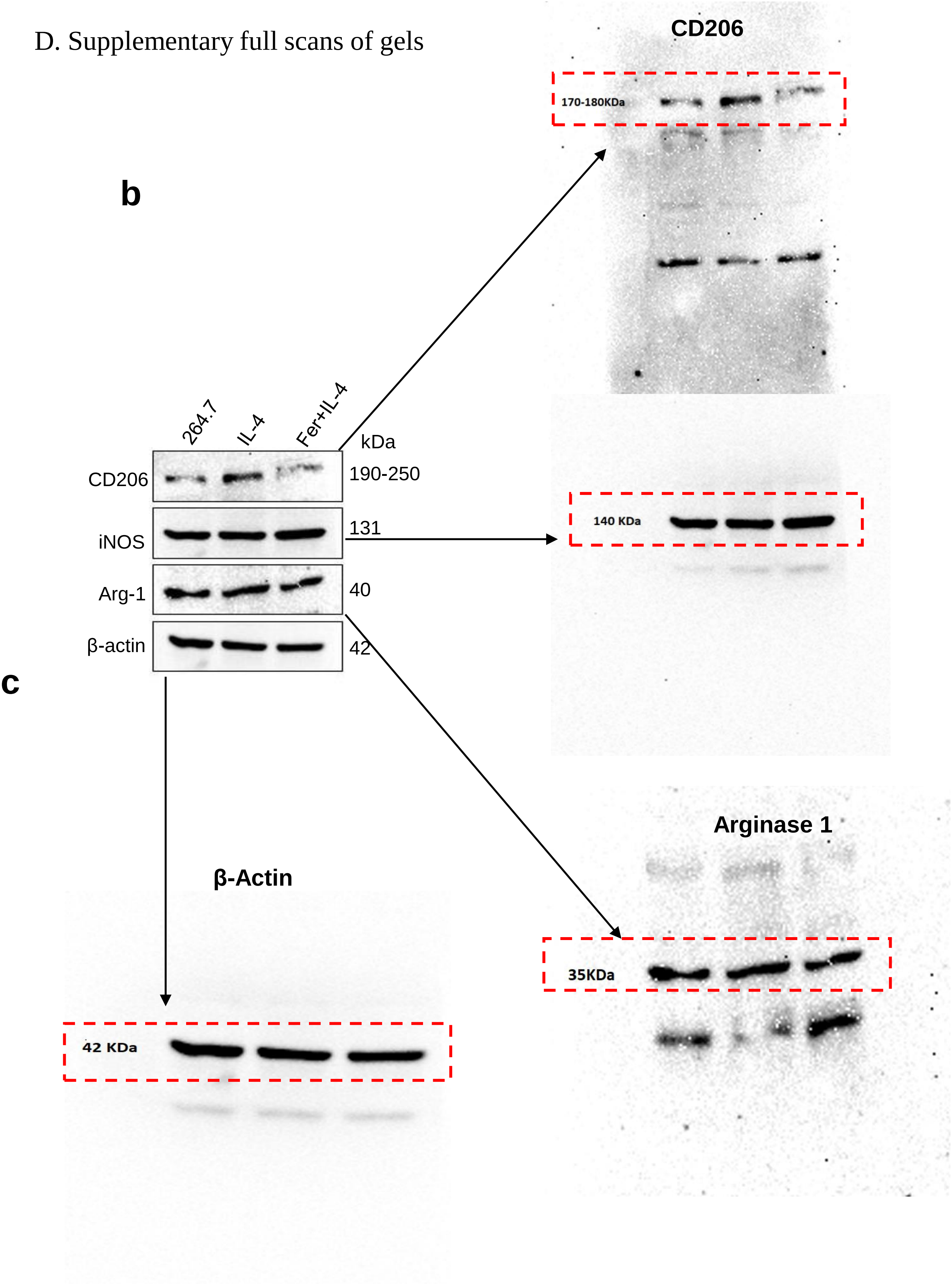

Supplementary Table 1

Complete list of flow cytometry reagents and antibodies

Antibody	Company	catalogue
MHCII APC cy7	Tonbo	25-5321-U025
CD206 PE-cy7	Invitrogen	25-2061-80
Ly-6G PerCP-cy5.5	Tonbo	65-1276-U025
Ly-6C APC	Biolegend	128015
F4/80 PE	Tonbo	50-4801-U025
CD11b redfluor 710	Tonbo	80-0112-U025
CD8a FITC	Tonbo	35-0081-U025
CD3 BV421	Biolegend	100227
CD45 VF 450	Tonbo	75-0451-U025
NOS2 APC-eFluor 780	Thermo Fisher	47-5920-80
CD16/CD32 (FC shield)	Tonbo	70-0161-U100
Ghost Dye violet 510	Tonbo	13-0870-T100
Foxp3/ Transcription Factor staining buffer kit	Tonbo	TNB-0607-vKIT
Transcription Factor fix/perm concentrate (4X)	Tonbo	TNB-1020-L050
Transcription Factor fix/perm Diluent (1X)	Tonbo	TNB-1022-L160
Flow cytometry perm buffer (10X)	Tonbo	TNB-1213-L150
UltraComp eBeads compensation beads	Invitrogen	01-2222-41
Tumor dissociation kit	Miltenyi Biotec	130-096-730
MACS SmartStrainers	Miltenyi Biotec	130-098-462
gentleMACS tubes	Miltenyi Biotec	130-093-237

Supplementary Table 2

List of reagents and antibodies

**Reagent**	**Company**	**Catalogue**
Ferrichrome	Sigma	F8014-5MG
Phagocytosis kit	Molecular probes	V-6694
CLI-095	Invitrogen	Tlrl-cli95
Iron stain kit	Abcam	Ab150674
**Antibody**	**Company**	**catalogue**
Arginase1 mouse	Santa cruz	Sc-47715
PD-L1 rabbit	Cell Signaling	13684S
CD206 rabbit	Cell signaling	12981S
F4/80 rabbit	Abcam	Ab6640
CK19 rabbit	Abcam	Ab52625
CD163 rabbit	Abcam	Ab182422
CD8	abcam	Ab203035
InVivoMAb anti-mouse PD-L1 (B7-H1)	BioXCell	BE0101
InVivoMAb rat IgG2b isotype control	BioXCell	BE0090

Supplementary Table 3

List of real time PCR primers

Gene	Forward	Reverse
Lcn2	TGGCCCTGAGTGTCATGTG	CTCTTGTAGCTCATAGATGGTGC
Fpn	TGGATGGGTCCTTACTGTCTGCTAC	TGCTAATCTGCTCCTGTTTTCTCC
PI3kγ	CGAGAGTGTCGTCACAGTGTC	TGTTCGCTTCCACAAACACAG
Mrc1	AGGGACCTGGATGGATGACA	TGTACCGCACCCTCCATCTA
PPARγ	TTCGATCCGTAGAAGCCGTG	TTGGCCCTCTGATGAGGA
MMP12	TGGGCTTCTCTGCATCTGTG	TTTGGTGACACGACGGAACA
IL-6	CTGCAAGAGACTTCCATCCAG	AGTGGTATAGACAGGTCTGTTGG
18S rRNA	GCAATTATTCCCCATGAACG	AGGGCCTCACTAAACCATCC
Nos2	TTCTGTGCTGTCCCAGTGAG	TGAAGAAAACCCCTTGTGCT
IL-12p40	AGCAGTAGCAGTTCCCCTGA	AGTCCCTTTGGTCCAGTGTG
TGF-β1	GGAGAGCCCTGGATACCAAC	CAACCCAGGTCCTTCCTAAA
Fizz1	CCCTTCTCATCTGCATCTCC	CTGGATTGGCAAGAAGTTCC
Ym1	TCTGGGTACAAGATCCCTGAA	TTTCTCCAGTGTAGCCATCCTT
CD11c	CTG GATAGCCTTTCTTCTGCTG	GCACACTGTGTCCGAACTCA
CD80	ACCCCCAACATAACTGAGTCT	TTCCAACCAAGAGAAGCGAGG
MHCII	GCGACGTGGGCGAGTACC	CATTCCGGAACCAGCGCA
IL-10	ATCGATTTCTCCCCTGTGAA	TGTCAAATTCATTCATGGCCT
Arg1	AGAGATTATCGGAGCGCCTT	TTTTTCCAGCAGACCAGCTT
TNF-α	CCACCACGCTCTTCTGTCTAC	AGGGTCTGGGCCATAGAACT
Hamp	TGTCTCCTGCTTCTCCTCCT	CTCTGTAGTCTGTCTCATCTGTTG
L-Ft	CGGAGGGTCAACATGCTATAA	AAG AGA CGG TGC AGA CTG GT
H-Ft	GCTGAATGCAATGGAGTGTG	CAGGGTGTGCTTGTCAAAGA
IL-1β	GCAACTGTTCCTGAACTCAACT	ATCTTTTGGGGTCCGTCAACT
PD-1	CAGCTTGTCCAACTGGTCG	GCTCAAACCATTACAGAAGGCG
MMP2	ACCTGAACACTTTCTATGGCTG	CTTCCGCATGGTCTCGATG
MMP9	GCAGAGGCATACTTGTACCG	TGATGTTATGATGGTCCCACTTG
