# Supplementary material for: Reprogramming of pancreatic adenocarcinoma immunosurveillance by a microbial probiotic siderophore
Source: Commun Biol. 2022 Nov 4;5:1181. doi: 10.1038/s42003-022-04102-4 (PMC9636404; doi:10.1038/s42003-022-04102-4)
Supplement: Supplementary file 3 — Description of Additional Supplementary Files [file 42003_2022_4102_MOESM3_ESM.pdf]

Description of Additional Supplementary Files

File name: Supplementary Data 1
Description: Raw data for Supplementary Figure 1.

File name: Supplementary Data 2
Description: Raw data for Supplementary Figure 2.

File name: Supplementary Data 3
Description: Raw data for Supplementary Figure 3.

File name: Supplementary Data 4
Description: Raw data for Supplementary Figure 4.

File name: Supplementary Data 5
Description: Raw data for Supplementary Figure 6.

File name: Supplementary Data 6
Description: Raw data for Supplementary Figure 7.
