# Supplementary material for: Reprogramming of pancreatic adenocarcinoma immunosurveillance by a microbial probiotic siderophore
Source: Commun Biol. 2022 Nov 4;5:1181. doi: 10.1038/s42003-022-04102-4 (PMC9636404; doi:10.1038/s42003-022-04102-4)
Supplement: Supplementary file 4 — Reporting Summary [file 42003_2022_4102_MOESM4_ESM.pdf]

nature portfolio

Corresponding author(s): Subhash Chauhan and Bilal Hafeez

Last updated by author(s): 07/18/2022

Reporting Summary

Nature Portfolio wishes to improve the reproducibility of the work that we publish. This form provides structure for consistency and transparency in reporting. For further information on Nature Portfolio policies, see our Editorial Policies and the Editorial Policy Checklist.

Statistics

For all statistical analyses, confirm that the following items are present in the figure legend, table legend, main text, or Methods section.

n/a	Confirmed	
☐	☒	The exact sample size (n) for each experimental group/condition, given as a discrete number and unit of measurement
☐	☒	A statement on whether measurements were taken from distinct samples or whether the same sample was measured repeatedly
☐	☒	The statistical test(s) used AND whether they are one- or two-sided<br>*Only common tests should be described solely by name; describe more complex techniques in the Methods section.*
☐	☒	A description of all covariates tested
☐	☒	A description of any assumptions or corrections, such as tests of normality and adjustment for multiple comparisons
☐	☒	A full description of the statistical parameters including central tendency (e.g. means) or other basic estimates (e.g. regression coefficient) AND variation (e.g. standard deviation) or associated estimates of uncertainty (e.g. confidence intervals)
☐	☒	For null hypothesis testing, the test statistic (e.g. F, t, r) with confidence intervals, effect sizes, degrees of freedom and P value noted<br>*Give P values as exact values whenever suitable.*
☒	☐	For Bayesian analysis, information on the choice of priors and Markov chain Monte Carlo settings
☒	☐	For hierarchical and complex designs, identification of the appropriate level for tests and full reporting of outcomes
☒	☐	Estimates of effect sizes (e.g. Cohen's d, Pearson's r), indicating how they were calculated

Our web collection on statistics for biologists contains articles on many of the points above.

Software and code

Policy information about availability of computer code

Data collection	No codes were used for data collection
Data analysis	Data analysis was done using GraphPad Prism, FlowJo, ImageJ

For manuscripts utilizing custom algorithms or software that are central to the research but not yet described in published literature, software must be made available to editors and reviewers. We strongly encourage code deposition in a community repository (e.g. GitHub). See the Nature Portfolio guidelines for submitting code & software for further information.

Data

Policy information about availability of data

All manuscripts must include a data availability statement. This statement should provide the following information, where applicable:
- Accession codes, unique identifiers, or web links for publicly available datasets
- A description of any restrictions on data availability
- For clinical datasets or third party data, please ensure that the statement adheres to our policy

Authors confirm that all relevant data are included in the main manuscript or supplementary files

Field-specific reporting

Please select the one below that is the best fit for your research. If you are not sure, read the appropriate sections before making your selection.

☒ Life sciences ☐ Behavioural & social sciences ☐ Ecological, evolutionary & environmental sciences

For a reference copy of the document with all sections, see nature.com/documents/nr-reporting-summary-flat.pdf

Life sciences study design

All studies must disclose on these points even when the disclosure is negative.

Sample size	The sample size and statistical methods were described in figure legends and methods
Data exclusions	For tumor in vivo studies, animals that did not have palpable tumors by begining of treatment were excluded from the study.
Replication	The replication was determined in each figure legend
Randomization	Research participants were randomly assigned to the sample groups
Blinding	The investigators were blinded to group allocation during data collection and analysis

Reporting for specific materials, systems and methods

We require information from authors about some types of materials, experimental systems and methods used in many studies. Here, indicate whether each material, system or method listed is relevant to your study. If you are not sure if a list item applies to your research, read the appropriate section before selecting a response.

Materials & experimental systems

n/a	Involved in the study	
☐	☒	Antibodies
☐	☒	Eukaryotic cell lines
☒	☐	Palaeontology and archaeology
☐	☒	Animals and other organisms
☒	☐	Human research participants
☒	☐	Clinical data
☒	☐	Dual use research of concern

Methods

n/a	Involved in the study	
☒	☐	ChIP-seq
☐	☒	Flow cytometry
☒	☐	MRI-based neuroimaging

Antibodies

Antibodies used	All antibodies used in this study are described in detail in supplementary files
Validation	Each product data sheet has listed the antibody standard validation on the manufacturer's website and relevant citations.

Eukaryotic cell lines

Policy information about cell lines

Cell line source(s)	Mouse pancreatic cancer cells lines UN-KC-6141 and luciferase-expressing Panc02 were a kind gift from Dr. Batra's laboratory at University of Nebraska Medical Center and Dr. Kazuaki Takabe's laboratory at the Roswell Park Cancer Institute in Buffalo, NY, respectively. UN-KPC 960 were a kind gift of Dr. Evan Glazer at UTHSC, Memphis TN. RAW264.7 murine macrophage cell line was obtained from American Type Culture Collection (ATCC, TIB-71, Manassas VA).
Authentication	Animals were handled in accordance with protocol approval by the UTHSC Institutional Animal Care and Use Committee (UTHSC-IACUC).
Mycoplasma contamination	Cell lines were negative for mycoplasma contamination.
Commonly misidentified lines (See ICLAC register)	None.

Animals and other organisms

Policy information about studies involving animals; ARRIVE guidelines recommended for reporting animal research

Laboratory animals	C57BL/6J mice were obtained from Jackson Laboratories (000664, Bae Harbor, ME). WT and TLR4-/- mice were a kind gift of Dr. Guoyun Chen (UTHSC, Memphis TN).
Wild animals	No wild animals were involved in the study.
Field-collected samples	All animals were housed in a temperature-controlled facility with a 12-h light/dark cycle and ad libitium access to food and water.
Ethics oversight	Animals were handled in accordance with protocol approval by the UTHSC Institutional Animal Care and Use Committee (UTHSC-IACUC).

Note that full information on the approval of the study protocol must also be provided in the manuscript.

Flow Cytometry

Plots

Confirm that:

☒ The axis labels state the marker and fluorochrome used (e.g. CD4-FITC).

☒ The axis scales are clearly visible. Include numbers along axes only for bottom left plot of group (a 'group' is an analysis of identical markers).

☒ All plots are contour plots with outliers or pseudocolor plots.

☒ A numerical value for number of cells or percentage (with statistics) is provided.

Methodology

Sample preparation	All sample preparation was described in details in the methods section of the manuscript.
Instrument	All instruments used for data collection were described in detail in the manuscript.
Software	All softwares used for data analysis were described in detail in the manuscript.
Cell population abundance	This study did not involve cell sorting.
Gating strategy	The gating strategy is provided in supplementary files.

☒ Tick this box to confirm that a figure exemplifying the gating strategy is provided in the Supplementary Information.
